# Influences of fatigue and anticipation on female soccer players’ biomechanical characteristics during 180° pivot turn: implication for risk and prevention of anterior cruciate ligament injury

**DOI:** 10.3389/fphys.2024.1424092

**Published:** 2024-08-29

**Authors:** Limin Zou, Xiaochun Zhang, Ziang Jiang, Xie Wu, Qiang Zhang

**Affiliations:** ^1^ College of Physical Educantion, Jinggangshan University, Ji’an, China; ^2^ Department of Medicine, Jinggangshan University, Ji’an, China; ^3^ Institute for Biomechanics, ETH Zürich, Zürich, Switzerland; ^4^ School of Exercise and Health, Shanghai University of Sport, Shanghai, China

**Keywords:** anterior cruciate ligament injury, fatigue, anticipation, turn, lower-limb biomechanics

## Abstract

**Introduction:**

Athletes’ capability to perform activities with body rotation could be weakened by fatigue accumulation. Making pivot turning in unanticipated scenarios after fatigue may greatly challenge athletes’ ability to adapt rational motion strategies, elevating the risk of anterior cruciate ligament (ACL) injury. This study aimed to investigate the effects of fatigue and anticipation on biomechanical risk factors of ACL injury during 180° pivot turns in female soccer players.

**Methods:**

Twenty-one female soccer players were selected as participants. The participants performed anticipated turning maneuver before the fatigue intervention. The participants sprinted along the runway, decelerated and planted their foot on the force plate, and then executed a 180° pivot turn. For unanticipated tests, the pivot turn was mixed with side/cross-cuts, which were indicated to the participant using a custom-designed light system. The tests were repeated by the participant after receiving a fatigue intervention. Lower-limb joint angles and moments were characterized. Peak ground reaction forces (GRFs) and GRF loading rates were determined. Two-way repeated measures analysis of variance was applied to examine the effects of fatigue and anticipation on the variables of interest.

**Results:**

Compared to the anticipated conditions, the approach speed was significantly lower in the unanticipated tests (*P* < 0.0001). Lower-limb kinematics showed varied angular patterns across conditions: greater hip joint variations in flexion, abduction, and internal rotation during unanticipated turns; consistent knee joint flexion and ankle plantarflexion with dorsiflexion observed mid-turn. Significant interactions (*P* = 0.023 to *P* = 0.035) between fatigue and anticipation influenced hip joint angles. Anticipation effects were notable at initial contact and peak ground reaction force, increasing hip, knee, and ankle joint angles (*P* < 0.0001 to *P* = 0.012). Participants showed consistent ground reaction force (GRF) patterns during pivot turns across fatigue and anticipation conditions, with the first peak occurring approximately 10% into the turn period. Significant interaction effects (*P* = 0.016) between fatigue and anticipation were observed for knee flex/extension moments at the first peak vertical GRF. Anticipation significantly increased first peak vertical (*P* < 0.0001), anteroposterior (*P* < 0.0001), and mediolateral (*P* < 0.0001) GRFs. Fatigue increased first peak vertical (*P* = 0.022), anteroposterior (*P* = 0.018), and mediolateral (*P* = 0.019) GRFs. Post-fatigue, participants exhibited reduced first peak GRFs and loading rates compared to pre-fatigue conditions, with higher rates observed in unanticipated turns (vertical GRF: *P* = 0.030; anteroposterior GRF: *P* < 0.0001).

**Conclusion:**

Female soccer players’ lower-limb Biomechanical characterization could be greatly affected by the change of anticipatory scenarios. With the associated increase of GRF, the risk of their ACL injury might be elevated. Fatigue affected female soccer players’ abilities on movement performances, but the interaction of these two factors could potentially weaken their knee’s functions during pivot turns. Cognitive training on unanticipated tasks may be important for rehabilitation training after ACL injury.

## Introduction

Epidemiological studies have shown that non-contact anterior cruciate ligament (ACL) injury is prevalent in the context of soccer matches (Boden et al., 2000; Collins et al., 2016). It was reported that approx. 70% of the ACL injuries in female soccer players occurred in the braking phase of pivot turns during competitions (Faude et al., 2005). Each player could complete over 700 times of movements with directional change in a soccer match (Nygaard Falch et al., 2019), which is often rapid in the circumstance of evading opponents or defense. From the biomechanical perspective, this may be attributed to the fact that knee joints possess great transversal rotations during pivot turn, with concomitant high loadings acted on the knee during body deceleration (Collins et al., 2016; Dos’ Santos et al., 2019; McLean et al., 2004). Therefore, investigating individuals’ lower-limb biomechanics in turn maneuvers is crucial for understanding the mechanisms of non-contact ACL injuries in soccer, particularly for the development of prevention and rehabilitation training programs.

Soccer players accumulate fatigue and encounter unanticipated scenarios during competition. Fatigue and anticipation have also been reported to greatly affect an individual’s lower-limb kinematics and kinetics during movements with directional change such as single-step cuts (Collins et al., 2016; Borotikar et al., 2008; McLean and Samorezov, 2009). However, the findings from previous studies are controversial. Some studies reported that fatigue and anticipation altered knee mechanics towards an increased risk of ACL injury in cut maneuvers (Collins et al., 2016; Borotikar et al., 2008). Other studies claimed that individuals adopted a protective strategy during post-fatigue (Cortes et al., 2014) or unanticipated cuts (Rolley et al., 2023), implying a potential reduction of ACL loading. Importantly, cut and pivot turn are very much different tasks, with dissimilar motion characteristics and demands (Greig, 2009). While individuals decelerate their body and change their direction simultaneously in a cut maneuver, the pivot turn could consist of a more prolonged and intensive braking phase before any body rotation happens. Therefore, people have argued that cut maneuvers cannot replicate the demands of a pivot turn which could more realistically represent a soccer task (Greig, 2009). Furthermore, a previous study has reported that compared to lateral cutting and landing movements, individuals exhibited smaller knee flexion and larger abduction at peak ground reaction forces (GRF) during pivot turns, with a higher knee joint loading that might potentially increase the risk of ACL injury (Cortes et al., 2011). Thus, individuals’ performances in pivot turns under neuromuscular perturbations cannot be fully explained by the findings from cutting tests, and should be investigated separately.

There is currently a lack of clear description on the influences of fatigue and anticipation to soccer players’ lower-limb biomechanics in pivot turn. During pivot turning, individuals may utilize complicated lower-limb movement coordination strategies to counteract the forward momentum of their upper body, and to connect with the subsequent body rotation (Newell, 1996; Newell and Slifkin, 1998). This substantially challenges the ability of their lower-limb joints, especially the knee, to resist overlarge rotations and loadings. Therefore, any fatigue on the lower-limb muscles may not only affect the muscular protection to the knee’s stability, but also change the overall motion control in the turn maneuvers. A previous study reported that soccer players could exhibited either protective or dangerous biomechanical behaviors during pivot turns after fatigue (Zago et al., 2021). Such inconsistency in their biomechanical performance may be explained by the compromise of the central and peripheral processing mechanisms under fatigue (Borotikar et al., 2008; Lorist et al., 2005). Importantly, when players are facing unanticipated tasks in a fatigue situation, they may likely experience the worst-case scenario for coordination of central and peripheral responses compared to either of these two situations alone. Considering fatigue and decision making in unanticipated task are commonly integrated in soccer matches, it is essential to investigate their interactions on the soccer players’ biomechanical performances in pivot turns, especially those factors associated ACL injury. Nevertheless, few studies have achieved this goal till now.

Studies have shown that female soccer players face 3–5 times higher risk of ACL injuries than their male counterparts, and this difference is largely attributed to differences in physiology and movement technique (Prodromos et al., 2007; Moses et al., 2012). Therefore, the purpose of this study was to test female soccer players’ biomechanical performances during anticipated and unanticipated turns, with a special focus on those variables matching the known ACL injury mechanism. This study also aimed to repeat the measurements after the participants received a fatigue drilling intervention, in order to study the integrated effects of anticipation and fatigue on their performances. The hypotheses of this study were: 1) Anticipation would greatly influent the kinematics and kinetics of the participants’ lower-limb joints in turn maneuvers. 2) The simultaneous presence of fatigue would further amplify their biomechanical changes, potentially resulting in even a higher risk of ACL injury.

## Methods

### Sample size calculation

The sample size calculation was performed using G*Power 3.0.10 software (Heinrich-Heine-Universität Düsseldorf, Germany). An effect size f of 0.27, derived from a pilot study, was used. The calculation aimed for a power of 0.80 and an alpha level of 0.05. The statistical method used for the calculation was a repeated measures ANOVA with within-subject factors. It was determined that a minimum of 21 participants would be required to detect significant effects.

### Participants

Participants were 21 female college soccer players, all of whom had a minimum of 5 years of training experience at the college level. All participants were national level athletes or above and had finished in the top three in national competitions. This high level of training background means that there may be significant differences in their motor skills, body control, and motor coping abilities, which is crucial for the interpretation of the results of the study of the effects of fatigue and anticipation on their motor performance. Recruitment was conducted through the professional networks of the research team members. The inclusion criteria included: 1) right dominant legs; 2) no cardiovascular or respiratory disease; 3) no history of lower limb surgery; 4) no injury to lower limbs in the past 6 months. This study was approved by the local Ethics Committee. Before measurement, participants were informed of the study aims and experiment protocol and then signed the informed consent form.

### Experimental setup

**FIGURE 1:**
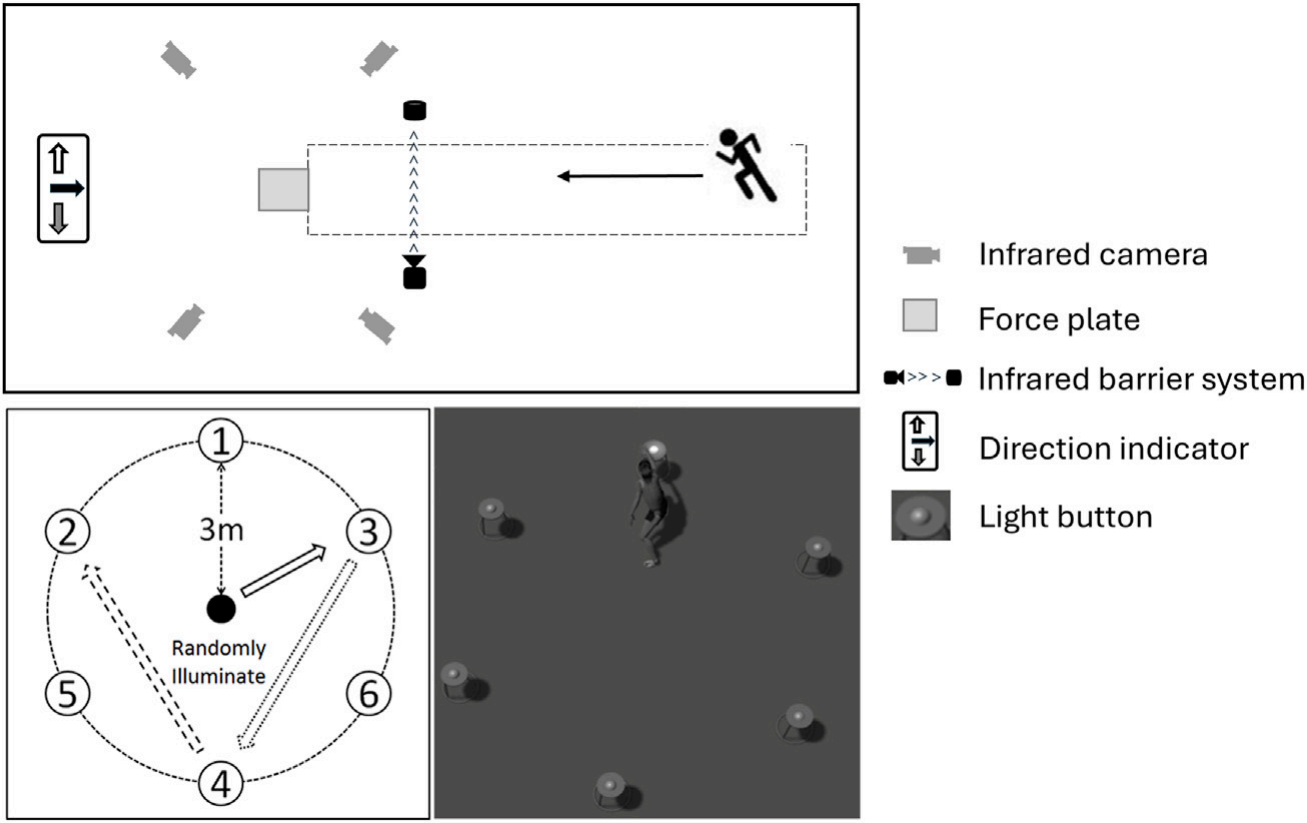
Illustration of pivot turn test and fatigue intervention protocol. Upper: The setup of the pivot test; bottom left: Scheme of participant’s movement directions during the fatigue intervention; bottom right: Illstration of the fatigue intervention.

A 16-camera motion analysis system (Vicon Motion Analysis Inc., Oxford, United Kingdom; 200 Hz) and a force plate (Kistler Instruments AG, Winterthur, Switzerland; 1,000 Hz) were used to capture 3D body motions and GRFs. Fourteen-millimeter reflective markers were bilaterally placed on specific anatomical landmarks, including the head of the first and fifth metatarsi, calcaneus, medial and lateral malleolus, medial and lateral femoral epicondyles, greater trochanter of the femur, anterior superior iliac spine, iliac crest, sacrum, sternum, xiphisternal joint, and acromion. Sixteen tracking markers were placed on the participant’s bilateral shanks and thighs. The test site is shown in Figure 1.

### Overall experiment protocol

Participants wore a spandex T-shirt, shorts, and sports shoes during the experiment. After 5-min warming up on a treadmill at 8 km/h, the participant was prepared for optical motion capture (MoCop) tests. The participant performed unanticipated and anticipated pivot turn tests (see section “Pivot turn test”), while their 3D body motions and GRFs were captured. The tests were performed in a randomized order with 30 s rest between the trials. Afterwards, the participant received fatigue interventions (see section “Fatigue protocol”). Once the target level of fatigue was met, the participant immediately repeated the unanticipated and anticipated turn tests.

### Fatigue protocol

The fatigue protocol consisted of a variety of movements including running, sidestep cutting, and jumping. Participants performed three countermovement jumps (CMJs) at a rhythm of 18 beats per minute regulated by a metronome. The peak jumping height calculated from the vertical displacements of a reflective marker on the participant’s sacrum was used as a reference to quantify their level of fatigue (Zago et al., 2021). Participants then stood in the center of a circular region with a diameter of 6 m, where six light buttons were placed on the circle’s edge. The buttons illuminated in a randomized order, and participants moved at their best to press the illuminated button, completing 25 presses followed by three CMJs. This cycle was repeated with 10-s breaks until a 30% reduction in jumping height was achieved, indicating the target level of fatigue. Participants spent an average of 39.2 ± 5.3 min performing 12.9 ± 3.5 fatigue drill cycles to achieve the target level of fatigue.

### Pivot turn test

Participants performed both anticipated and unanticipated 180° pivot turn tests on a 9-m-long and 2-m-wide runway with a force plate embedded at its center. An infrared barrier system was placed 2 m before the force plate to accurately capture the moment of initial contact (IC). In the anticipated condition, participants were informed of the movement direction (pivot turn) beforehand and instructed to execute the turn using their right leg.

In the unanticipated condition, cutting movements were introduced to mimic the unpredictable nature of real-game scenarios. Upon reaching the infrared barrier system, a custom-designed light system was triggered, indicating the required movement direction just before the participants reached the force plate. The direction was signaled using arrows: a left arrow for a side cut (45°), a right arrow for a cross-cut (90°), and a downward arrow for a pivot turn (180°). The order of light gate activation was randomized using a random number generator (www.random.org), ensuring that participants could not prepare in advance.

To perform the pivot turn, participants had to decelerate from their sprint, plant their right foot on the force plate, and execute a 180° turn using their right leg as the pivot. A test was considered successful if the participant completed the turn within the designated area without stepping outside the force plate, maintained balance throughout the motion, and executed the turn fluidly without hesitation or excessive pausing. Trials were repeated if these criteria were not met to ensure consistency and reliability in the collected performance data. Three successful trials were recorded for the unanticipated and anticipated conditions.

### Data processing

The biomechanical analysis suite, Visual3D (C-Motion Inc., Germantown, MD, United States), was used to compute participants’ 3D kinematic and kinetic variables. Marker trajectories and analog signals from the force plate were low-pass filtered using a Butterworth 4th-order bidirectional filter with cutoff frequencies of 20 Hz and 100 Hz, respectively. The 3D angular variables were defined using a Cardan sequence (X-Y-Z), with the rotation order as flexion/extension (*X*-axis), abduction/adduction (*Y*-axis), and internal/external rotation (*Z*-axis). The GRF was expressed in three directions: vertical (V-GRF), anteroposterior (AP-GRF), and mediolateral (ML-GRF). A right-hand rule was applied to determine the polarity of the joint angles and GRFs.

The pivot turn period was defined from the IC of the participant’s foot on the force plate (vertical force over 10N) to when the foot left the force plate (vertical force below 10N). Approach speed was calculated as the sprinting speed at IC. Lower-limb joint angles and GRFs throughout the pivot turn period were time-normalized as a percentage of the turn period and then plotted. The first peak GRFs in the three directions were determined and normalized to each participant’s body weight (BW). The GRF loading rate was calculated as the quotient of the first peak V-GRF and the time to reach this peak from IC.

Knee joint moments at the first peak V-GRF were characterized and normalized to each participant’s body mass (kg). Joint angles of the hip, knee, and ankle at IC were extracted, and knee joint angles at the first peak V-GRF were characterized. Joint angles and moments in the sagittal, frontal, and transverse planes were reported to provide a comprehensive analysis of joint movements and their potential relevance to non-contact ACL injuries.

Joint angles at IC and peak GRF were selected based on their relevance to ACL injury mechanisms, as indicated by previous studies (Zago et al., 2021). Initial contact is a critical phase where the body’s alignment and initial load distribution can influence injury risk. The first peak V-GRF represents the maximum vertical force experienced, capturing the peak loading conditions that are critical for understanding knee mechanics and potential injury mechanisms during pivot turns. Analyzing these points provides insights into the biomechanical demands and injury risk factors associated with these specific moments during dynamic movements.

### Statistical analysis

The results were averaged from outcomes of the three trials in each condition and were presented as mean ± standard deviation (SD). Repeated measures analysis of variance (ANOVA) was conducted to evaluate the kinematic and kinetic variables of interest, with the factors being fatigue (pre-fatigue vs. post-fatigue) and anticipation (anticipated vs. unanticipated). Assumptions of normality and sphericity for Repeated Measures ANOVA were examined. Normality was assessed using the Shapiro-Wilk test, and sphericity was evaluated using Mauchly’s test. If the assumption of sphericity was violated, the Greenhouse-Geisser correction was applied. In cases where the assumption of normality was violated, the non-parametric Aligned Rank Transform test was used as an alternative. When significant interactions between fatigue and anticipation were found, simple main effects were analyzed to determine the specific effects of anticipation at each level of fatigue and the effects of fatigue at each level of anticipation. Effect size (ES) was calculated using the method of Cohen’s d and classified as: small 0.2–0.49, medium 0.5–0.79, and large >0.8 (Yeung, 2014). Statistical analysis was performed using SPSS software (version 28.0; SPSS Inc., Chicago, IL, United States). An alpha level of 0.05 was set for all statistical tests.

## Result

### Participant demographics

The demographic data of the participants are as follows: age: 18.4 ± 1.4 years; height: 1.66 ± 0.05 m; mass: 60.1 ± 5.5 kg; training experience: 9.2 ± 1.6 years. Initially, 21 participants were recruited, all of whom completed the study with no dropouts.

### Approach speed

Table 1 presents the approach speeds before the pivot turn motion. There was no significant interaction between fatigue and anticipation on approach speed (Table 1). However, a significant main effect of anticipation was found (F_1,20_ = 54.241, *P* < 0.001, d = 0.812), with participants sprinting significantly slower in unanticipated turns compared to anticipated conditions. No significant main effect of fatigue was observed.

**TABLE 1:** Approach speed before the pivot turn motion.

Variables	Pre-fatigue		Post-fatigue		Fatigue	Anticipation	Interaction
	Anticipated	Unanticipated	Anticipated	Unanticipated	*P*	*P*	*P*
Approach speed (m/s)	2.60 ± 0.26	2.38 ± 0.22	2.53 ± 0.19	2.37 ± 0.26	0.100	<0.001*	0.336

*Bold value indicates statistical significance (*P* < 0.05).

### Lower limb kinematics

**FIGURE 2:**
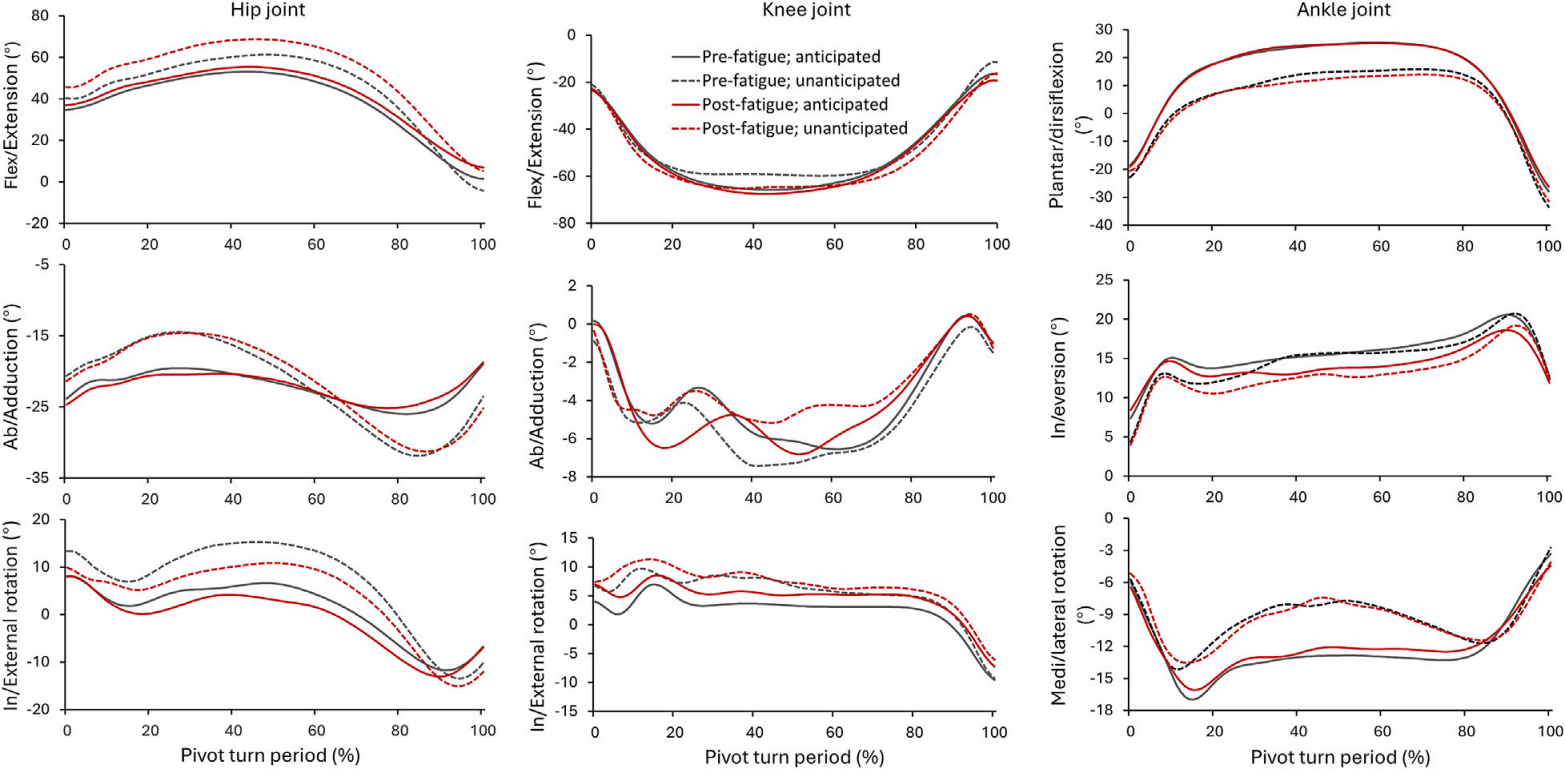
Angular variations of hip, knee, and ankle joints throughout the pivot turn period in unanticipated and anticipated tests before and after fatigue. The joint angles were expressed in three planes: sagittal (flex/extension), frontal (ab/adduction), and transverse (in/external rotation) planes.

The angular patterns of the lower-limb joints in the pivot turns under different conditions were shown in Figure 2. The participant’s hip joint possessed flexion and abduction during the entire turn period, with a change from internal to external rotation. It could be observed that compared to their performance during anticipated turns, the participants exhibited greater angular variations in their hip joints in all the three anatomical planes during unanticipated turns. In addition, the participant’s knee joint possessed flexion, abduction, and internal rotation (0% to approx. 95%) during the turn period. Their knee angular variations were similar among different fatigue and anticipation conditions. Finally, they exhibited ankle plantarflexion during the first and last 10% of the turn period, but dorsiflexion during the middle phase. Meanwhile, they exhibited ankle inversion and lateral rotation. They ankle angular variations in the sagittal and transverse planes were smaller during unanticipated turns compared to anticipated turns.

Table 2 details the kinematics of lower-limb joints at IC and at the first peak vertical ground reaction force (vGRF). Significant interactions between fatigue and anticipation were observed for hip in/external rotation angle at IC (F_1,20_ = 6.075, *P* = 0.023, d = 0.160) and at first peak vGRF (F_1,20_ = 5.017, *P* = 0.035, d = 0.163). For the main effects, anticipation significantly affected multiple variables at IC. During unanticipated turns, the participants exhibited significantly greater hip flexion angle (F_1,20_ = 12.301, *P* = 0.002, d = 0.518), smaller hip abduction angle (F_1,20_ = 13.025, *P* = 0.002, d = 0.477), greater hip internal rotation angle (F_1,20_ = 16.634, *P* = 0.001, d = 0.488), greater ankle plantarflexion angle (F_1,20_ = 10.393, *P* = 0.004, d = 0.359), and smaller ankle inversion angle (F_1,20_ = 32.434, *P* < 0.001, d = 0.556). At the first peak vGRF, the participants possessed significantly larger hip flexion angle (F_1,20_ = 8.299, *P* = 0.009, d = 0.437), smaller hip abduction angle (F_1,20_ = 10.425, *P* = 0.004, d = 0.370), larger hip internal rotation angle (F_1,20_ = 22.939, *P* < 0.001, d = 0.488), smaller knee flexsion angle (F_1,20_ = 7.606, *P* = 0.012, d = 0.420), larger knee internal rotation angle (F_1,20_ = 34.694, *P* < 0.001, d = 0.707), larger ankle plantarflexion angle (F_1,20_ = 60.131, *P* < 0.001, d = 1.237), smaller ankle inversion angle (F_1,20_ = 30.575, *P*< 0.001, d = 0.444), and smaller ankle lateral rotation angle (F_1,20_ = 10.024, *P* = 0.005, d = 0.235).

**TABLE 2:** Lower-limb joint kinematics at IC and the first peak ground reaction force in pre- and post-fatigue states during anticipated and unanticipated pivot turns.

Time point	Variables	Pre-fatigue		Post-fatigue		Fatigue	Anticipation	Interaction
		Anticipated	Unanticipated	Anticipated	Unanticipated	*P*	*P*	*P*
IC	Hip flex/extension angle (°)	34.7 ± 11.1	40.3 ± 18.8	36.9 ± 11.3	45.6 ± 12.9	0.164	0.002*	0.469
	Hip ab/adduction angle (°)	−23.8 ± 7.6	−20.6 ± 6.6	−24.7 ± 7.1	−21.3 ± 6.1	0.365	0.002*	0.914
	Hip in/external rotation angle (°)	8.0 ± 7.9	13.4 ± 8.6	8.1 ± 5.5	9.9 ± 7.1	0.123	0.001*	0.023*
	Knee flex/extension angle (°)	−22.6 ± 6.2	−20.9 ± 10.9	−23.2 ± 5.1	−22.8 ± 5.0	0.265	0.378	0.594
	Knee ab/adduction angle (°)	0.2 ± 4.7	−0.9 ± 4.6	−0.0 ± 4.1	−0.4 ± 4.3	0.777	0.140	0.311
	Knee in/external rotation angle (°)	4.0 ± 8.8	6.7 ± 8.6	7.0 ± 9.2	7.4 ± 8.5	0.222	0.166	0.178
	Ankle plantar/dorsiflexion angle (°)	−18.6 ± 8.9	−22.8 ± 8.3	−19.1 ± 6.6	−20.6 ± 7.2	0.535	0.004*	0.052
	Ankle in/eversion angle (°)	7.4 ± 6.6	4.4 ± 6.3	8.4 ± 6.3	4.0 ± 7.3	0.712	<0.001*	0.062
	Ankle medi/lateral rotation angle (°)	−5.9 ± 4.4	−5.7 ± 4.8	−6.4 ± 5.4	−5.2 ± 4.8	0.967	0.158	0.186
First peak vGRF	Hip flex/extension angle (°)	37.4 ± 10.7	41.4 ± 18.4	40.0 ± 11.9	48.2 ± 13.6	0.087	0.009*	0.347
	Hip ab/adduction angle (°)	−21.5 ± 8.5	−18.8 ± 7.3	−22.3 ± 7.5	−19.4 ± 6.9	0.435	0.004*	0.898
	Hip in/external rotation angle (°)	3.9 ± 7.7	10.9 ± 7.8	3.4 ± 6.8	7.3 ± 7.2	0.101	<0.001*	0.035*
	Knee flex/extension angle (°)	−35.3 ± 8.1	−30.7 ± 12.8	−36.6 ± 7.3	−33.9 ± 5.2	0.073	0.012*	0.476
	Knee ab/adduction angle (°)	−3.4 ± 4.1	−3.6 ± 4.7	−3.7 ± 4.1	−4.1 ± 4.6	0.461	0.544	0.898
	Knee in/external rotation angle (°)	0.2 ± 6.9	4.4 ± 7.5	2.5 ± 5.5	7.6 ± 6.0	0.058	<0.001*	0.456
	Ankle plantar/dorsiflexion angle (°)	1.9 ± 12.0	−9.9 ± 7.8	2.4 ± 10.9	−10.0 ± 7.9	0.889	<0.001*	0.665
	Ankle in/eversion angle (°)	13.8 ± 6.5	10.7 ± 6.6	14.0 ± 5.8	11.4 ± 6.1	0.576	<0.001*	0.695
	Ankle medi/lateral rotation angle (°)	−10.7 ± 6.3	−9.4 ± 6.0	−10.4 ± 5.8	−9.0 ± 5.5	0.731	0.005*	0.911

IC, initial contact; vGRF, vertical ground reaction force, *Bold values indicate statistical significance (*P* < 0.05).

### Lower limb kinetics

**FIGURE 3:**
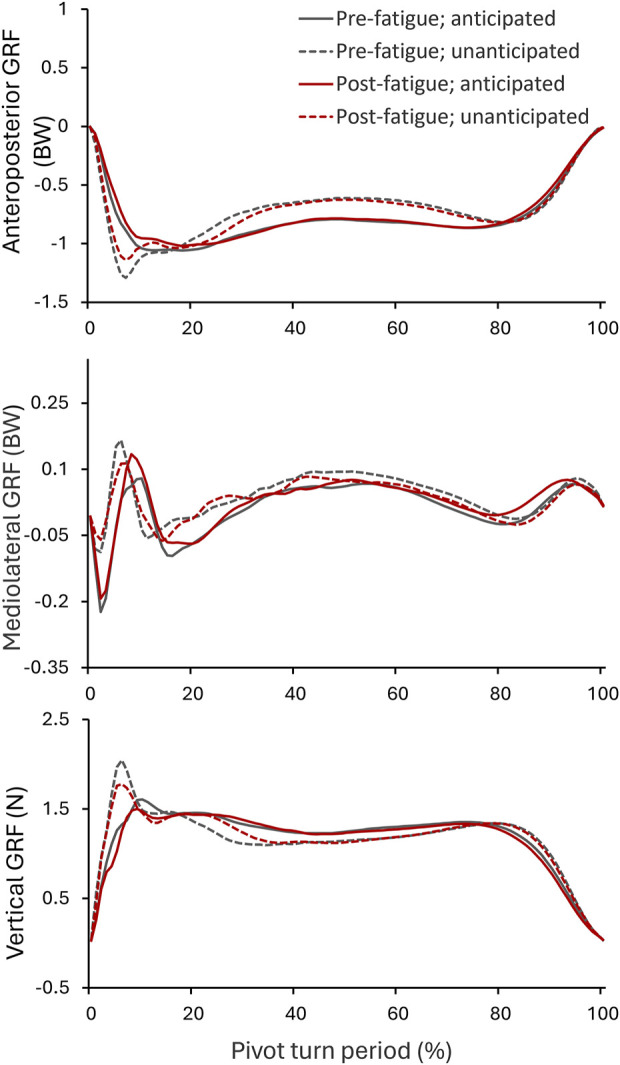
Variations of ground reaction forces throughout the pivot turn period in unanticipated and anticipated tests before and after fatigue. The ground reaction forces were expressed in three directions: anteroposterior, mediolateral, and vertical vectors.

The patterns of GRF during the pivot turns under different conditions were shown in Figure 3. The GRF variations were similar among different fatigue and anticipation conditions. The first peak GRF occurred shortly after IC, at approx. 10% of the turn period.

Table 3 provides detailed information on GRFs and knee joint moments. A significant interaction between fatigue and anticipation was found for the knee flex/extension moment at first peak vertical GRF (F_1,20_ = 6.979, *P* = 0.016, d = 0.686). Simple effects analysis indicated that before the fatigue intervention, the knee flexion moment was significantly greater during unanticipated turns compared to anticipated turns (*P* = 0.006), but this difference was not significant after the fatigue intervention (*P* = 0.154). In terms of changes in joint kinetics, the main effect of anticipation was signficiant on knee ab/adduction and in/external rotation moment at first peak vertical GRF. Compared to their perforamces during anticipated turns, the participant exhibitied significantly smaller knee adduction moment (F_1,20_ = 54.831, *P* < 0.001, d = 1.244) and smaller internal rotation moment (F_1,20_ = 39.211, *P* < 0.001, d = 1.358) during unanticipated turns.

**TABLE 3:** Ground reaction forces and knee joint moments in pre- and post-fatigue states during anticipated and unanticipated pivot turns.

Variables	Pre-fatigue		Post-fatigue		Fatigue	Anticipation	Interaction
	Anticipated	Unanticipated	Anticipated	Unanticipated	*P*	*P*	*P*
First peak vertical GRF (BW)	2.26 ± 0.39	2.76 ± 0.44	2.17 ± 0.48	2.55 ± 0.45	0.022*	<0.001*	0.181
First peak anteroposterior GRF (BW)	−1.33 ± 0.22	−1.53 ± 0.21	−1.27 ± 0.20	−1.45 ± 0.23	0.018*	<0.001*	0.710
First peak mediolateral GRF (BW)	−0.27 ± 0.11	−0.19 ± 0.10	−0.24 ± 0.09	−0.14 ± 0.76	0.019*	<0.001*	0.623
Loading rate of vertical GRF (BW/s)	73.37 ± 35.76	109.15 ± 51.08	68.48 ± 38.61	87.33 ± 30.34	0.030*	0.002*	0.150
Loading rate of anteroposterior GRF (BW/s)	27.52 ± 15.94	38.48 ± 14.55	23.15 ± 17.33	38.66 ± 15.39	0.282	<0.001*	0.193
Knee flex/extension moment (N•m/kg)	−0.00 ± 1.20	−0.83 ± 1.32	−0.59 ± 1.13	−0.46 ± 1.24	0.432	0.193	0.016*
Knee ab/adduction moment (N•m/kg)	2.82 ± 0.82	1.56 ± 1.16	2.56 ± 0.89	1.46 ± 0.87	0.235	<0.001*	0.562
Knee in/external rotation moment (N•m/kg)	0.49 ± 0.20	0.22 ± 0.26	0.50 ± 0.19	0.21 ± 0.22	0.978	<0.001*	0.710

BW, body weight; GRF, Ground Reaction Force; *Bold values indicate statistical significance (*P* < 0.05).

For main effects, anticipation significantly affected the first peak vertical GRF (F_1,20_ = 27.996, *P* < 0.001, d = 0.989), first peak anteroposterior GRF (F_1,20_ = 48.128, *P* < 0.001, d = 0.883), and first peak mediolateral GRF (F_1,20_ = 32,621, *P* < 0.001, d = 0.232). Fatigue showed significant effects on the first peak vertical GRF (F_1,20_ = 6.147, *P* = 0.022, d = 0.337), first peak anteroposterior GRF (F_1,20_ = 6.644, *p* = 0.018, d = 0.318), and first peak mediolateral GRF (F_1,20_ = 6.521, *P* = 0.019, d = 0.103). The main effects of fatigue and anticipation on the loading rate of GRF were also significant. The loading rate to the first peak vertical GRF was signficantly smaller in post-fatigue turns than that in pre-fatigue turns (F_1,20_ = 5.478, *P* = 0.030, d = 0.336), and was significantly larger in unanticipated turns than that in anticipated turns (F_1,20_ = 6.245, *P* = 0.002, d = 0.584). Similarly, the loading rate to the first peak posterior GRF was significantly larger during unanticipated turns compared to anticipated turns (F_1,20_ = 18.569, *P*< 0.001, d = 0.835).

## Discussion

Concurrence fatigue and unanticipated tasks can cause deviations in soccer players’ performances during real compitations (Rolley et al., 2023; Benjaminse et al., 2019). Their integrative influence on individuals’ lower-limb kinematics and kinetics in rapid pivot turns, especially those associated with non-contact ACL injury, however remain less known. This study characterized soccer players’ biomechanical performances during anticipated and unanticipated pivot turns, both before and after fatigue. In general, this study revealed that anticipation affected the participants’ lower-limb biomechanics towards an increased risk of ACL injury during pivot turning. The results also indicated that the participants’ movement abilities were weakened by fatigue. Finally, this study revealed significant interactions between fatigue and anticipation factors particularly for the hip and knee biomechanical performances, underscoring the complex interplay of biomechanical adjustments athletes undergo to cope with cognitive challenges during turning tasks.

### Kinematic analysis

Participants exhibited pronounced changes in hip joint kinematics, particularly in unanticipated turns. Specifically, there was increased hip flexion and abduction angles, alongside greater variability in internal and external rotation angles. This suggests that unanticipated situations necessitate rapid and extensive hip movements to accommodate abrupt changes in movement demands. Moreover, increased hip internal rotation at IC during unanticipated turns is consistent with previous research (Clemens and Pew, 2023; Guex et al., 2012). Such internal rotation is associated with heightened knee joint abduction moments during directional changes (McLean et al., 2005), potentially compromising the efficacy of quadriceps and hamstrings in countering external knee abduction forces (Delp et al., 1999; Besier et al., 2003). Additionally, participants demonstrated smaller hip adduction rotation angles at IC in unanticipated turns, contrasting with findings of larger hip abduction angles in similar conditions (Norte et al., 2020; Imwalle et al., 2009). Greater hip abduction has been linked to increased knee abduction and potential ACL injury risk (Imwalle et al., 2009). Analysis of knee joint dynamics revealed notable differences during anticipated and unanticipated turns. Participants exhibited less knee flexion but increased internal rotation at peak vGRF in unanticipated turns (Besier et al., 2001). These findings align closely with previous systematic reviews indicating increased knee flexion and internal rotation angles in unanticipated single-leg cuts (Almonroeder et al., 2015). Reduced knee flexion angles can lead to greater anterior tibial shear force, a direct contributor to ACL injury (Aghdam et al., 2022; Padua and DiStefano, 2009; Favre et al., 2016; Podraza and White, 2010). Furthermore, decreased knee flexion diminishes the hamstring’s ability to counteract anterior shear forces, thereby potentially increasing ACL loading (Padua and DiStefano, 2009). The ankle joint exhibited significant movement pattern changes throughout the turn phases. Notably, there was dorsiflexion at the middle and plantar flexion at the beginning and end of turns (Favre et al., 2016). Compared to anticipated turns, non-anticipatory turns showed less angular variation in sagittal and transverse sections, particularly in dorsiflexion and inversion (Podraza and White, 2010). This underscores the critical influence of anticipation on ankle stability and movement patterns during rapid changes in direction. An interaction between fatigue and anticipation was observed, particularly concerning hip angles at peak vGRF. This interaction suggests that fatigue exacerbates the challenge of predicting and executing movements, especially under unanticipated conditions. Such heightened cognitive demands may alter lower limb postures, potentially increasing the risk of ACL injuries during the braking phase of unanticipated turns (Vassalou et al., 2016). These findings have important clinical implications for injury prevention and rehabilitation strategies in athletes. The observed movement patterns and joint dynamics highlight specific vulnerabilities during unanticipated maneuvers, particularly concerning ACL injury risks associated with hip and knee joint mechanics.

### Kinetic analysis

In this study, anticipatory effects on GRFs during pivot turns were also notable, with participants exhibiting higher peak vertical and posterior GRFs, alongside elevated GRF loading rates during unanticipated turns. This rapid peak loading upon ground contact indicates a reactive adaptation to unexpected movement demands. Our results align with previous research (Meinerz et al., 2015) but differ from others (Rolley et al., 2023), likely due to variations in tested movements across studies. During the braking phase of pivot turns, individuals must decelerate from sprinting speed to zero. In unanticipated scenarios, decision-making to change direction may lead to less effective coordination of body motions, resulting in a more rigid landing strategy. Increased GRFs and loading rates during rigid landings are recognized risk factors for lower limb injuries (Weinhandl and O'Connor, 2017), particularly non-contact ACL injuries (Podraza and White, 2010; Cacolice et al., 2020; Bates et al., 2013). Larger vertical GRFs increase knee loading, potentially affecting stability (Bates et al., 2013), while higher posterior GRFs may augment anterior shear forces on the tibia, potentially straining the ACL (Cacolice et al., 2020; Badiola-Zabala et al., 2020). In summary, the simultaneous alterations in knee kinetics and peak GRFs highlight an increased risk of ACL injuries during unanticipated tasks. Fatigue predominantly influenced participants’ GRFs rather than joint kinematics. Post-fatigue, participants exhibited significantly lower GRFs across all components, with reduced vertical GRF loading rates. These findings are consistent with prior studies (Cortes et al., 2014; Zhang et al., 2021) reporting decreased GRFs following fatigue induction during side-step cuts and jump landings. Reduced GRFs post-fatigue may stem from diminished central nervous system excitability and muscle force production (Quammen et al., 2012; Tornero-Aguilera et al., 2022). The nature of fatigue, whether local or systemic, varied depending on protocols and participants, uniformly impairing muscle force generation and movement control, thereby diminishing overall performance. For instance, Hollman et al. (2012) found that hip extensor fatigue resulted in reduced hip flexion during jump-landing tasks among women. This underscores the nuanced effects of localized fatigue on specific joint dynamics, warranting further investigation in pivot turns.

This study revealed several key findings regarding the impact of fatigue on biomechanical parameters during pivot turns. Before fatigue induction, participants exhibited larger knee flexion moments during unanticipated turns. This observation suggests that individuals utilized knee flexors to stabilize the knee joint as the GRF vector likely passed anterior to the knee during this phase. This proactive knee flexion may serve to protect the knee from excessive loading and potential injury. Following fatigue induction, there was a slight reduction in knee flexion moment during unanticipated turns. This decline could indicate a diminished ability of knee flexors to adequately protect the knee joint under fatigue conditions. Despite this reduction in knee flexion moment, the study noted an increase in GRF during unanticipated turns post-fatigue. This discrepancy raises concerns as reduced knee flexion may compromise knee joint stability, potentially increasing susceptibility to ACL injuries. The knee joint relies on coordinated muscle actions to manage GRF effectively, and fatigue-induced alterations in muscle function could compromise this protective mechanism. Moreover, the findings underscore the complex interplay between fatigue, knee biomechanics, and injury risk during dynamic movements like pivot turns. While the study did not directly measure kinematic changes, the observed alterations in knee flexion moments highlight a critical aspect of knee joint dynamics affected by fatigue. These results align with existing literature suggesting that fatigue impairs neuromuscular control, leading to suboptimal movement patterns and increased injury risk (Fortes et al., 2024).

## Limitations

This study had several limitations. Our lab-based fatigue protocol, while standardized, did not fully replicate real-match conditions. Future research should use soccer-specific tasks to better simulate sport-related fatigue. Additionally, it was difficult to separate the effects of neuromuscular and mental fatigue on performance. Future studies should isolate these effects and their interactions with anticipatory factors. Lastly, our participants were healthy soccer players. Future research should test fatigue and anticipation effects on individuals with ACL injuries and use the findings to develop targeted rehabilitation programs to prevent ACL re-tearing. Using only peak jump height to assess fatigue without considering other types of fatigue (e.g., cognitive fatigue), potentially leading to bias on the evaluation of fatigue levels. In addition, the study was conducted with uninjured soccer players. Individuals with a history of ACL injuries should be considered in the future. Finally, this study did not explore the effects of different athletic tasks on fatigue and turning performances, and the small sample size may limit the generalizability of the results.

## Conclusion

In unanticipated situations, lower limbs exhibited distinct kinematic and kinetic patterns during pivot turns compared to anticipated situations. These deviations were associated with an elevated risk of ACL injury. Fatigue might impair female soccer players’ limb stability production and movement control, affecting their dynamic performances. However, its effect on the risk of their ACL injury was vague. Training to enhance female soccer players’ cognitive skills may potentially reduce the risk of non-contact ACL injuries in unanticipated scenarios during competitions, particularly to players rehabilitated from ACL ruptures.

## Data Availability

The original contributions presented in the study are included in the article/supplementary material, further inquiries can be directed to the corresponding author.
